# Prevalence, satisfaction and preference of tooth shades and their correlation with age, gender and skin shade: A cross sectional study

**DOI:** 10.12688/f1000research.146428.2

**Published:** 2025-03-05

**Authors:** Prashanth Bajantri, Tanay Chawda, Srikant Natarajan, Alisha Ono, Thilak Shetty, Shobha Rodrigues, Umesh Pai, Mahesh M, Sharon Saldanha, Puneeth Hegde, Sandipan Mukherjee, Ann Sales, Vignesh Kamath

**Affiliations:** 1 Prosthodontics Crown and Bridge, Manipal College of Dental Sciences Mangalore, Manipal Academy of Higher Education, Manipal, Karnataka, 576104, India; 2 Oral pathology, Oral pathology, Manipal College of Dental Sciences, Manipal Academy of Higher Education, Mangalore, Karnataka, 576104, India

**Keywords:** Shade selection, VITA Tooth-Guide 3D Master, esthetics

## Abstract

Shade selection is one of the most important factors that determines the satisfaction of the patient with the dental prosthesis and thus the overall success of the treatment. Conventional methods of shade selection require the presence of existing teeth or past dental/photographic records. Despite the presence of these records, shade selection continues to be challenging, subjective and arbitrarily based on the patient’s age and gender especially if the visual method is employed. This study aims to assess the prevalence, satisfaction, and preference of tooth shades in a cross section of the local Indian population, investigating their correlation with age, gender, and skin color in a diverse sample of 120 participants visiting the outpatient department of this Institution.

**Methods:**

A total of 120 participants, 60 males and 60 females, categorized into four age (n=15): groups 20-30, 30-40, 40-50, & 50-60 yrs were recruited randomly and evaluated visually using the VITA 3D Master in the study. Participants were also asked about their satisfaction with existing tooth shades and their preferences.

**Results:**

The most common existing tooth shade was Value 2 (52.5%).

Statistical analysis using Chi-square tests revealed that Value 2 was the most desired shade among males and females alike, particularly in the age group of 41 to 50 years (p < 0.001).

Shade 2L1.5 was the most prevalent (n=26), with Value 2 (59.16%) being the most sought-after shade, followed by Shade 2L1.5 (n=21), Shade 2M1 (n=18), Shade 1M1 (n=15), and Shade 2R1.5 (n=14).

Existing tooth shades of Value 0 were the least common.

The highest satisfaction with existing shades was observed in males aged 41 to 50 (n=15), followed by males aged 51 to 60 (n=14) and females aged 31 to 40 (n=12) and 41 to 50 (n=12). Significant differences in satisfaction were noted among males (p < 0.001).

**Conclusions:**

This study reveals a consistent and strong preference among both males and females for tooth shades classified as Value 2, following the VITA Tooth-Guide 3D Master shade guide. This universal preference suggests the relevance and acceptance of this shade category across diverse patient groups.

## Introduction

Facial esthetics have become important in modern society as they seem to define one’s character. Dental appearance is one of the main features that determines the attractiveness of our face and plays a key role in our social interactions. ^1^ It has been found that the most important factors that influence the perception of beautiful teeth are age, gender, and skin color. ^2^^,^^3^

Shade selection is an important procedure for providing patients with an esthetic restoration that harmoniously blends with the patient’s existing dentition. ^4^ The selection of teeth with proper shade positively influences the patient’s aesthetic perception and improves prosthesis acceptance. ^5^ Shade selection is a compound phenomena and requires knowledge of the physics, physiology and psychology of colour and is both an art and a science requiring in depth knowledge, accurate clinical judgement and perception on the part of the dentist.

The selection of artificial teeth to replace missing natural teeth is a relatively straightforward procedure when natural anterior teeth remain. Technology advances have made available a wide variety of shade guides for use in patients with natural teeth. However, the choice of tooth shade is problematic for edentulous individuals with no preextraction records. ^6^

Various factors such as age, sex, and skin color have been proposed as aids for artificial tooth shade selection. ^7^ Some dentists have even suggested the color of hair and eye color, but most authors favor the use of facial skin tone as a guide for tooth shade selection. ^8^

There is limited scientific information on the relationship between facial skin complexion and tooth color in the Indian population. This lack of knowledge may impact the ability of prosthodontists to provide better esthetics to the patient.

The perception among dentists is that individuals with darker skin color have lighter shades of teeth. This perception is commonly explained by the illusion of a greater contrast between skin color and tooth shade. ^3^

The age of the patient was found to have a definite relationship with the tooth shade value, which has been corroborated by many studies that have shown darker tooth shade values with an increase in age and vice versa. ^3^^,^^6^

Gender is another factor significantly associated with tooth shade values. Men are more likely to have darker tooth shade values, whereas women of the same age group are more likely to have lighter tooth shade values. ^9^

Various studies have been conducted to establish a relationship between age, sex, and skin color in individuals with tooth shades, but contrasting results have been reported. One reason for the varying results can be attributed to the ethnic origin of the study samples. ^8^^,^^9^

Individual studies conducted by Ajayi et al. (2011), Albashaireh et al., Hamamci et al. (2009), and Afshar MK et al. (2019) had reported satisfaction with dental appearance of 79.4% in Nigeria, ^10^ 67.6% in Jordan, ^11^ 71.1% in Turkey, ^12^ and 47.2% in Malaysia. ^13^

A study conducted by Maghaireh et al. (2016) found that most people were not satisfied with their tooth color, and the sought-after treatment was tooth whitening. They also reported that women are significantly more likely to seek cosmetic and orthodontic restorations. ^14^

In a study by Tin-Oo (2011), satisfaction with tooth color was significantly lower in women, and tooth whitening was the most preferred treatment. ^15^

Research in the field of esthetics and shade matching has predominantly been conducted in Western populations. However, with increasing dental awareness and demand for esthetics in developing countries, such as the Indian subcontinent, research in the field of esthetics based on the local population has become the need of the hour.

This study aimed to determine the prevalence, satisfaction, and preference of tooth shades and their correlation with sex, age, and skin color.

The null hypothesis was that there would be no difference in the prevalence, preference, and satisfaction of tooth shades between the age class, gender, and skin color patterns.

## Methods

### Study setting

A cross section of about 120 subjects visiting the outpatient Department of Prosthodontics and Crown and Bridge, of this Institution were randomly recruited for the study Informed consent was obtained under a protocol reviewed and approved by the Institutional board.

### Pilot study and sample size calculation

A pilot study was conducted which showed a prevalence of satisfaction of 56.52% with an alpha of 5 % the corresponding z value was Z=-1.96.

Using the formula n=(Z(α/2)/d) 2^ “p(1-p)” the minimum percentage difference to be deemed clinically significant at 10%, the minimum sample size required would be 95 for the study. A method of matched quota sampling was employed to recruit an equal number of participants in each age group and sex. For the easy divisibility of the groups, a sample size of 120 was selected.

### Eligibility criteria

120 participants were recruited in this study using the following inclusion criteria:

1) All six maxillary anterior teeth (canine-to-canine) (13, 12, 11, 21, 22, 23) should be present.
2) Teeth should have no external discoloration or staining
3) All the 6 maxillary anterior teeth should have similar shades

### Grouping

The 120 randomly recruited participants were divided into eight groups based on their age and gender, as follows:

**Table:** 

Group 1: Males & females in the age group 20-30 yrs
Group 2: Males & females in the age group 30-40 yrs
Group 3: Males & females in the age group 40-50 yrs
Group 4: Males & females in the age group 50-60 yrs

The color of their teeth was visually evaluated using the 3D Master shade Guide.

### Procedure

*Questionnaire*

The participants were then asked to complete a questionnaire on their age, gender, and ethnic origin. They were then shown a face mirror and asked if they were satisfied with the color of their upper front teeth.

*Shade selection*

The Investigator underwent the Ishihara test to check for negative color blindness

Shade selection was done under natural lighting from the northern sky

Bright colour surroundings were avoided and female patients were asked to remove lipstick before commencing the shade selection

A light blue colour screen was used as a background during shade selection

A thorough oral prophylaxis was performed and shade selection was done under moist conditions at the operators eye level at an arms length

A squint test was performed and the least conspicuous color was designated as the most appropriate shade of the tooth

**Figure 1.**
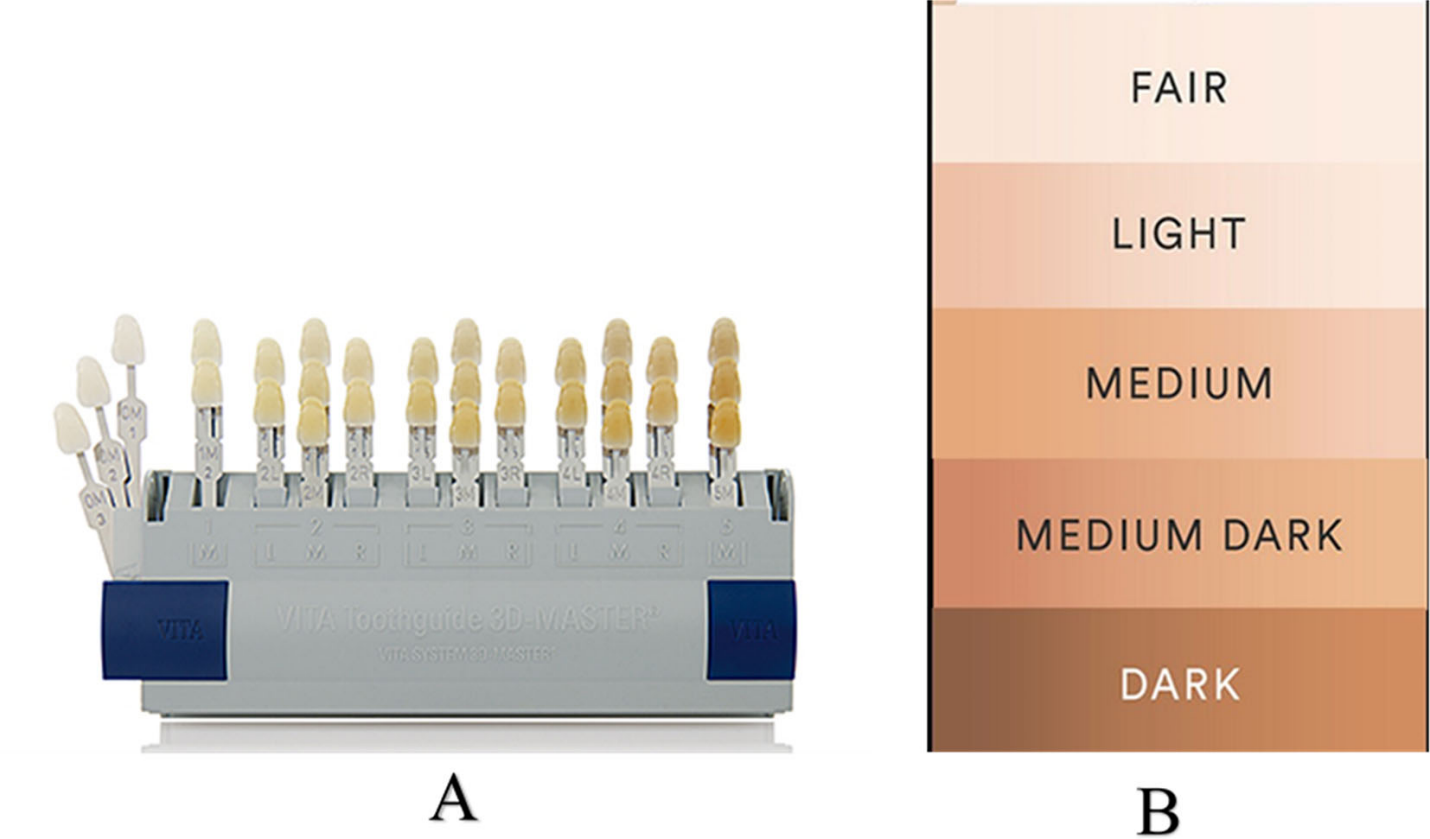
A - VITA Tooth-Guide 3D Master. B - L'Oreal skin shade guide.

The investigators thus selected the tooth shade that best matched the participants shade using a shade guide (3D Master, VITA Zahnfabrik H. Rauter GmbH & Co. KG Postfach 1338 D-79704 Bad Säckingen), as per manufacturer instructions ( Figure 1A).

If the test subjects were not satisfied with their existing tooth shade, they were guided to find the preferred tooth shade using the “VITA Tooth-Guide 3D Master” shade guide.

The participants’ skin color was recorded using the L’Oreal skin shade guide ( Figure 1B).

### Data management and statistical analysis

After collecting the data from all 120 participants, statistical analysis was done using χ ^2^ test to correlate tooth shade, skin color, age and gender. Odds ratios were calculated using a logistic regression. Statistical significance was considered at 95% confidence interval, and p values less than 0.05 were considered significant. IBM SPSS Statistics version 20.0 was used for these statistical calculations.

## Results

A total of 120 participants were grouped based on their age and sex to evaluate the prevalence, satisfaction, and preference of tooth shade and their correlation with age, sex, and skin shade using questionnaires along with the VITA Tooth-Guide 3D Master shade guide and L’Oreal skin shade guide.

### Existing tooth shades

As can be seen in Table 1, the most common existing among the participants was Value 2 (52.5%) of VITA Tooth-Guide 3D Master shade. Shade 2L1.5 (n=26) was the most common, followed by 3L1.5 (n=14), 2M1 (n=12), 2R1.5 (n=10), and 2M2 (n=9).

**Table 1.** Percentages of existing value of tooth shade and the wanted value of tooth shade by the participants.

	Percentage of participants who had the value of shade	Percentage of participants who wanted the value of shade
Value 0	0%	3.33%
Value 1	8.33%	20%
Value 2	52.5%	58.33%
Value 3	36.66%	17.5%
Value 4	2.5%	0.83%

The least common existing tooth shades were of value 0, with no participants showing any existing shade.

### Desired tooth shades

The most commonly desired tooth shade was of value 2 (58.33%). Shade 2L1.5, the most commonly preferred tooth shade (n=21), followed by 2M1 (n=18), 1M1 (n=15), 2R1.5 (n=14), and 1M2 (n=9).

Chi-square tests revealed that value 2 was the most preferred shade among males and females equally in the age group of 41–50 years. The value 2 was statistically significant in the higher age group (p<0.001) ( Table 2).

**Table 2.** Comparison of shade wanted by males and females of different age groups and their chi-square analysis.

Chi-Square Tests				
Age Group		Value	df	P value (<0.05 is significant)
20-30	Pearson Chi-Square	4.419	4	.352
	N of Valid Cases	30		
31-40	Pearson Chi-Square	5.559	3	.135
	N of Valid Cases	30		
41-50	Pearson Chi-Square	**<0.001**	2	1 **<0.001**
	N of Valid Cases	30		
51-60	Pearson Chi-Square	2.274	2	.321
	N of Valid Cases	30		

### Satisfaction

Among the shades present, highest satisfaction was observed with people having shades of value 1 (90%), followed by values 2 (82.25%), value 3 (44.44%), and value 4 (33.33%).

**Figure 2.**
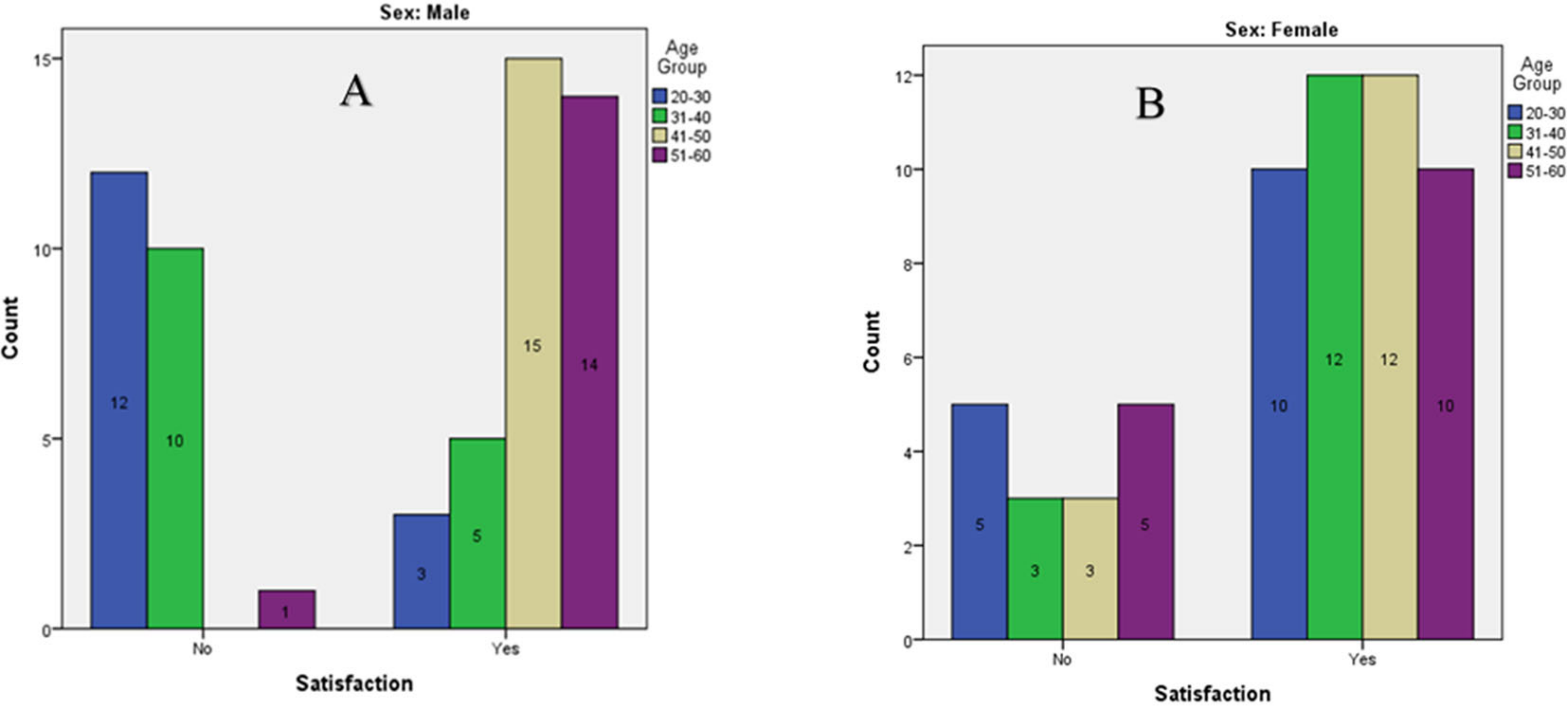
Bar graph of Satisfaction of tooth shade among participants of different age groups. A) Males. B) Females.

As shown in Figure 2, the highest satisfaction with existing shade was seen in males aged 41–50 years (n=15), followed by males aged 51–60 years (n=14), and females aged 31–40(n=12) and 41–50 (n=12). The lowest satisfaction was observed in males aged 20-30 (n=12), followed by males aged 31–40 years (n=10), and females aged 20-30 (n=5).

The differences in satisfaction with tooth shades among the males were statistically significant (p<0.001) ( Table 3).

**Table 3.** Comparison of satisfaction with existing shades of teeth among males and females of different age groups and their chi-square analysis.

Chi-Square Tests				
Sex		Value	df	P value (<0.05 is significant)
Female	Pearson Chi-Square	1.364	3	.714
	N of Valid Cases	60		
Male	Pearson Chi-Square	31.798	3	**<0.001**
	N of Valid Cases	60		

Sex			Age Group				Total
			20-30	31-40	41-50	51-60	
Female	Satisfaction	No	5	3	3	5	16
		Yes	10	12	12	10	44
	Total		15	15	15	15	60
Male	Satisfaction	No	12	10	0	1	23
		Yes	3	5	15	14	37
	Total		15	15	15	15	60

### Skin colour

No statistically significant correlations or differences were found between participants with different skin colour concerning shade present, shade wanted, and satisfaction with existing shades.

## Discussion

Color is a visual perception that results from light interacting with objects and the eye's photoreceptors. The perceived colour is also influenced by psychological precondition of an individual. Most people associate lighter and brighter teeth with beauty, however it is essential that the colour has to be in harmony with the face especially when a single esthetic indirect restoration is planned. To ensure good esthetics,the attributes of colour namely, hue, chroma and value must be correctly chosen. Amongst these attributes Value is most important since a small variation in chroma and hue will not be noticeable if value blends. ^16^

Methods of dental shade selection can be broadly categorized into visual and digital methods. Visual methods, popularly used include the use of stocks and custom shade guides. Digital methods include digital cameras, color-measuring software, colorimeters, spectrophotometers, and intraoral scanners. Despite developments in dental shade selection methods, shade selection remains a challenge that affects esthetic outcomes. ^17^

This challenge is augmented when patients are edentulous and lack any prior photographic or dental records of the shade of their original teeth, and conventional dental shade selection tools cannot be used.

According to protocol, the age, sex, and complexion of the patient should be taken into consideration while choosing the dental shade for completely edentulous patients. There are several recommendations for selecting the shape and size of artificial teeth, which are supported by rationale and empirical data. ^18^^–^^21^

However, shade selection for completely edentulous patients follows vague and broad criteria and is therefore more subjective and arbitrary. These add complexities to complete dentures and full-mouth rehabilitation. ^18^^–^^23^

This study aimed to explore the prevalence , satisfaction and preference of tooth shade values and their correlation with age, gender, and skin color. A diverse, but convenient sample of 120 was s randomly recruited to represent a cross section of the local population.

The current study demonstrated that the age and gender of the patient affect their satisfaction with existing dental shades and their shade preferences. No correlation was observed between skin shade and existing or desired shade in the observed test subjects. These observations are similar to previous studies by Esan TA et al. and Al Dwairi et al. ^24^^,^^25^

However the results are in contrast to a similar study performed. ^26^

The difference in the results may be extrapolated to the difference in the population studied as well as the methodology employed.

The results of this study showed that people become more satisfied with their shade as age advances. As age advances,people tend to become more involved in solving daily issues and are more concerned with greater challenges in life rather than their tooth colour.

In addition, shades of value 2 were the most prevalent and the most prefered shade by males and females as compared to shades of other values. The study found a significant relationship between the shade of teeth and age groups. 80% of the participants in age group of 30 – 40 & 40 – 50 years were satisfied with their shades, however in the younger age group only 67% were satisfied with their existing shade. As people age, their teeth tend to become darker, which is likely due to the formation of secondary dentin. The study also revealed that most patients irrespective of their age preferred teeth with Value 2, which is consistent with the value of the general population in the Indian subcontinent. This may be because shades of lower value are in harmony with their skin texture and appear most naturalised. As against this approximately 10% of the participants preferred brighter teeth. The perspective of what beauty entails differs from person to person. Some percentage of people presume brighter teeth, though not confluent with its surroundings to be more aesthetic. In contrast to the above result about 11.2% of patients preferred shades of lower value confirming that the perception of aesthetics varies between individuals.

Extrapolating the above results to clinical settings, patients desirous of brighter teeth are candidates who may consider tooth whitening, laminate veneers or crowns.

The most wanted/liked shades by the test subjects were 2L1.5 (17.5%), 2M1 (15%), 1M1 (12.5%), 2R1.5 (11.67%), 1M2 (7.5%), 2M2 (6.67%), and 2L2.5 (5.83%).

In the age group of 20 to 30 years, 53.3% of the female participants had shades of value 2, while 40% had shades of value 3. 66.7% were satisfied with their existing shade. 46.7% liked shades of value 2 and 26.7% liked value 3.

53.3% of the male participants had shades of value 2, while 33.3% had shades of value 3. 20% were satisfied with their existing shade. 46.7% liked value 2 and 33.3% liked value 1.

In the age group of 30 to 40 years, 46.7% of the female participants had shades of value 3, while 40% had shades of value 2. Eighty% were satisfied with their existing shade. 53.3% liked value 2, and 26.7% liked value 3.

46.7% of the male participants had shades of value 2, while 46.7% had shades of value 3. 33.3% were satisfied with their existing shade. Sixty% liked value 2, and 33.3% liked value 1.

In the age group of 40 to 50 years, 53.3% of the female participants had shades of value 2, while 40% had shades of value 3. Eighty% were satisfied with their existing shade. 73.3 % liked value 2, and 20% wanted to liked value 3.

66.7% of the male participants had shades of value 2, while 20% had shades of value 3. 80% were satisfied with their existing shade.

In age group 50 to 60, 40% of the female participants had shades of value 3, while 40% had shades of value 4. 66.7% were satisfied with their existing shade. 53.3% liked value 2, and 26.7% liked value 1.

66.7% Of the male participants had shades of value 2, while 26.7% had shades of value 3. 93.3% were satisfied with their existing shade. 73% liked value 2 and 20% liked value 3.

Males in the higher age groups were more satisfied with their teeth than younger age groups and females. This was also observed in studies conducted by Tin-Oo et al. and Maghaireh et al. ^14^^,^^15^

## Conclusions

Within the limitations of this study, the following conclusions can be drawn:

1. Satisfaction with existing shade increases in males, especially in the age group of 41–50 years.
2. The most desired tooth shades by both males and females were of value 2, as per the VITA Tooth-Guide 3D Master shade guide.

### Limitations of the study

1. In this study, participants with different skin shades were not selected. Light shade (n=5), fair shade (n=14), medium shade (n=45), Medium Dark shade (n=37), or dark shade (n=19). This can influence the results with respect to the relationship between skin shade and the satisfaction of existing tooth shades, along with tooth shades preferred by the participants of different skin shades.
2. A larger sample size may reveal further correlations between the hue, value, and chroma of existing and preferred tooth shades.

#### Key points

1. In the case of the unavailability of dental photographic records in edentulous patients, age and sex can act as a guide for shade selection.
2. If the patient or dentist is unsure about the shade of complete dentures and full-mouth rehabilitations, shades of value 2 can be selected, as they are the most liked shades according to the present study.
3. Older men may prefer darker shades of teeth, whereas women of all ages may prefer lighter shades.

#### Ethical considerations

All observations were performed in conformity with the ethical standards of the Institutional Ethics Committee, Manipal College of Dental Sciences Mangalore, after receiving approval from the committee (ref:22007 dated:12/02/2022). Each individual assigned to participate in the study gave written informed consent to participate in the study.

## Data Availability

Figshare: prevalence, satisfaction and preference of tooth shades and their correlation with age, gender and skin shade: a cross sectional study, DOI: https://doi.org/10.6084/m9.figshare.24903114.v3. ^27^ This project contains the following:
• Raw data Data are available under the terms of the Creative Commons Attribution 4.0 International license (CC-BY 4.0). Figshare: prevalence, satisfaction and preference of tooth shades and their correlation with age, gender and skin shade: a cross sectional study, DOI: https://doi.org/10.6084/m9.figshare.24903114.v3. ^27^ This project contains the following:
• Questionnaire Data are available under the terms of the Creative Commons Attribution 4.0 International license (CC-BY 4.0). Figshare: prevalence, satisfaction and preference of tooth shades and their correlation with age, gender and skin shade: a cross sectional study, DOI: https://doi.org/10.6084/m9.figshare.24903114.v3. ^27^ This project contains the following:
• STROBE CHECK Data are available under the terms of the Creative Commons Attribution 4.0 International license (CC-BY 4.0).

## References

[1] Samorodnitzky-Naveh GR Geiger SB Levin L : Patients’ satisfaction with dental esthetics. *J. Am. Dent. Assoc.* 2007;138:805–808. 10.14219/jada.archive.2007.0269 17545270

[2] Jahangiri L Reinhardt SB Mehra RV : Relationship between tooth shade value and skin color: an observational study. *J. Prosthet. Dent.* 2002;87:149–152. 10.1067/mpr.2002.121109 11854669

[3] McAndrew R : *Contemporary Fixed Prosthodontics.* 3rd ed.Vol.29. Sage;2014 Dec; pp.328–329. 10.1093/ortho/29.4.328-a

[4] Azad A Ahmad S Zia M : Relationship of age, gender and skin tone to shades of permanent maxillary central incisors. *Pak. Oral Dent. J.* 2007;27:119–125.

[5] Moon RJ Millar BJ : Dental Aesthetics: A Study Comparing Patients’ Own Opinions with Those of Dentists. *Open J. Stomatol.* 2017 Apr 17 [cited 2022 Jan 6];07:225–233. 10.4236/ojst.2017.74016

[6] Marcucci B : A shade selection technique. *J. Prosthet. Dent.* 2003;89:518–521. 10.1016/S0022-3913(03)00076-3 12806332

[7] Hammad IA : Intrarater repeatability of shade selections with two shade guides. *J. Prosthet. Dent.* 2003;89:50–53. 10.1067/mpr.2003.60 12589286

[8] Zarb G Bolender CL : *Prosthodontic treatment for edentulous patients: complete dentures and implant-supported prostheses.* 12th ed. St. Louis: Mosby Inc.;2004; pp.190–207.

[9] Bauer J Vasilache I Schlegel AK : Esthetics and Psyche—Part 1 Assessment of the Influence of Patients’ Perceptions of Body Image and Body Experience on Selection of Existing Natural Tooth Color. *Int. J. Prosthodont.* 2012;25:36–43. 22259794

[10] Ajayi EO : Dental aesthetic self-perception and desire for orthodontic treatment among school children in Benin City, Nigeria. *Nig. Q. J. Hosp. Med.* 2011;21(1):45–49. 21913541

[11] Albashaireh ZSM Alhusein AA Marashdeh MM : Clinical assessments and patient evaluations of the esthetic quality of maxillary anterior restorations. *Int. J. Prosthodont.* 2009;22(1):65–71. 19260431

[12] Hamamci N Baaran G Uysal E : Dental Aesthetic Index scores and perception of personal dental appearance among Turkish university students. *Eur. J. Orthod.* 2009 Apr;31(2):168–173. 10.1093/ejo/cjn083 19126820

[13] Afshar MK Eskandarizadeh A Torabi M : Patient Satisfaction with Dental Appearance and Related FactorsA Cross Sectional Study. *J. Evol. Med. Dent. Sci.* 2019;8:3569–3574. 10.14260/jemds/2019/771

[14] Maghaireh GA AIzraikat H Taha NA : Satisfaction with Dental Appearance and Attitude toward improving Dental Esthetics among Patients attending a Dental Teaching Center. *J. Contemp. Dent. Pract.* 2016;17:16–21. 10.5005/jp-journals-10024-1796 27084857

[15] Tin-Oo MM Saddki N Hassan N : Factors influencing patient satisfaction with dental appearance and treatments they desire to improve aesthetics. *BMC Oral Health.* 2011 Feb 23;11:1–8. 10.1186/1472-6831-11-6 21342536 PMC3059271

[16] Hassel AJ Nitschke I Dreyhaupt J : Predicting tooth color from facial features and gender: results from a white elderly cohort. *J. Prosthet. Dent.* 2008;99:101–106. 10.1016/S0022-3913(08)60025-6 18262010

[17] Tabatabaian F Beyabanaki E Alirezaei P : Visual and digital tooth shade selection methods, related effective factors and conditions, and their accuracy and precision: A literature review. *J. Esthet. Restor. Dent.* 2021;33:1084–1104. 10.1111/jerd.12816 34498789

[18] Sharry JJ : *Complete denture prosthodontics.* 3rd ed. New York: McGraw-Hill;1974.

[19] Johnson DL Stratton RJ : *Fundamentals of removable prosthodontics.* Chicago: Quintessence;1980; pp.289–307.

[20] Pound E : Applying harmony in selecting and arranging teeth. *Dent. Clin. N. Am.* 1962;6:241–258.

[21] Landa LS : Practical guidelines for complete denture esthetics. *Dent. Clin. N. Am.* 1977;21:285–298. 10.1016/S0011-8532(22)03210-4 321275

[22] Heartwell CM Rahn AO : *Syllabus of complete dentures.* 3rd ed. Philadelphia: Lea & Febiger;1980; pp.293–306.

[23] Zarb GA Bolender CL Hickey JC : *Boucher’s prosthodontic treatment for edentulous patients.* 10th ed. St Louis: CV Mosby Co;1990; pp.330–342.

[24] Esan TA Olusile AO Akeredolu PA : Factors Influencing Tooth Shade Selection for Completely Edentulous Patients. *J. Contemp. Dent. Pract.* 2006;7:80–87. 10.5005/jcdp-7-5-80 17091143

[25] Al-Dwairi Z Shaweesh A Kamkarfar S : Tooth shade measurements under standard and nonstandard illumination and their agreement with skin color. *Int. J. Prosthodont.* 2014;27:458–460. 10.11607/ijp.3826 25191889

[26] Vadavadagi SV Kumari KV Choudhury GK Prevalence of Tooth Shade and its Correlation with Skin Colour - A Cross-sectional Study. J. Clin. Diagn. Res. 2016 Feb;10(2): ZC72-4. 10.7860/JCDR/2016/16918.7324 PMC4800657 27042590

[27] Bajantari P : Prevalence, satisfaction, and preference of tooth shades and their correlation with age, gender, and skin shade: a cross-sectional study.Dataset. *figshare.* 2023. 10.6084/m9.figshare.24903114.v3 PMC12203042 40585878

