# Prevalence, satisfaction and preference of tooth shades and their correlation with age, gender and skin color : A cross sectional study

**DOI:** 10.12688/f1000research.146428.3

**Published:** 2025-07-14

**Authors:** Prashanth Bajantri, Tanay Chawda, Srikant Natarajan, Alisha Ono, Thilak Shetty, Shobha Rodrigues, Umesh Pai, Mahesh M, Sharon Saldanha, Puneeth Hegde, Sandipan Mukherjee, Ann Sales, Vignesh Kamath

**Affiliations:** 1 Prosthodontics Crown and Bridge, Manipal College of Dental Sciences Mangalore, Manipal Academy of Higher Education, Manipal, Karnataka, 576104, India; 2 Oral pathology, Oral pathology,Manipal College of Dental Sciences Mangalore, Manipal Academy of Higher Education, Karnataka, 576104, India

**Keywords:** Shade selection, VITA Tooth-Guide 3D Master, esthetics

## Abstract

Shade selection is a crucial factor influencing patient satisfaction with dental prostheses and the overall success of treatment. Conventional visual methods of shade selection are inherently subjective, regardless of the availability of dental records or photographs. This study aims to assess the prevalence, satisfaction, and preference of tooth shades in a cross-section of the local Indian population and to evaluate their correlation with age, gender, and skin color in a diverse sample of 120 participants visiting the outpatient department of this Institution.

**Methods:**

A total of 120 participants, comprising 60 males and 60 females across four age groups (20-30, 30-40, 40-50, and 50-60 years), were visually evaluated using the VITA 3D Master shade guide. Participants also indicated their satisfaction with their current tooth shade and their preferred shade.

**Results:**

Value 2 was the most prevalent existing shade (52.5%) and the most preferred across all groups, especially among individuals aged 41-50 years (p < 0.001). Satisfaction was highest among males aged 41–50 years. No significant association was found between skin color and tooth shade preference or satisfaction.

**Conclusions:**

The results suggest that shades in the Value 2 category are universally preferred, offering a useful reference for shade selection in clinical practice.

## Introduction

Facial esthetics play a significant role in modern society, influencing perceptions of character and social interactions. Dental appearance is a major component of facial esthetics, and shade selection is essential for creating restorations that blend harmoniously with natural dentition. ^1^ Factors such as age, gender, and skin color significantly affect perceptions of dental esthetics. ^2^^,^^3^

Proper tooth shade selection enhances the esthetic appeal and acceptance of dental prostheses. ^4^^,^^5^ The process is both an art and a science, involving an understanding of color physics, visual perception, and clinical judgment. While selecting teeth is straightforward in the presence of natural anterior teeth, it becomes challenging in edentulous patients without pre-extraction records.

The selection of artificial teeth to replace missing natural teeth is a relatively straightforward procedure when natural anterior teeth remain. Technology advances have made available a wide variety of shade guides for use in patients with natural teeth. However, the choice of tooth shade is problematic for edentulous individuals with no preextraction records. ^6^

Visual methods using shade guides remain the most common approach, though they are subjective and influenced by numerous external and individual factors. Digital techniques like spectrophotometry offer more objective assessments but are not always feasible in all clinical settings. ^7^^,^^8^

Studies have explored the relationship between age, gender, and skin tone with tooth shade, with inconsistent results due to variations in sample demographics and methodologies. In India, there is a lack of comprehensive data correlating skin tone with tooth shade, affecting the esthetic outcomes in prosthodontic treatment. The perception among dentists is that individuals with darker skin tones often appear to have lighter tooth shades. This is typically attributed to the visual illusion created by the higher contrast between skin color and tooth shade. ^3^

Age has been consistently linked to tooth shade value, with numerous studies reporting that tooth shades tend to darken with increasing age due to changes such as secondary dentin formation and enamel thinning. ^3^^,^^6^

Gender also plays a significant role, with men generally exhibiting darker tooth shades than women of the same age group, who are more likely to have lighter shades. ^9^

Although several studies have attempted to correlate age, gender, and skin color with tooth shade, the results have been inconsistent. These discrepancies are often attributed to variations in ethnic backgrounds and sampling methods across different populations. ^8^^,^^9^

Individual studies conducted by Ajayi et al. (2011), Albashaireh et al., Hamamci et al. (2009), and Afshar MK et al. (2019) had reported satisfaction with dental appearance of 79.4% in Nigeria, ^10^ 67.6% in Jordan, ^11^ 71.1% in Turkey, ^12^ and 47.2% in Malaysia. ^13^

A study conducted by Maghaireh et al. (2016) found that most people were not satisfied with their tooth color, and the sought-after treatment was tooth whitening. They also reported that women are significantly more likely to seek cosmetic and orthodontic restorations. ^14^

In a study by Tin-Oo (2011), satisfaction with tooth color was significantly lower in women, and tooth whitening was the most preferred treatment. ^15^

Research in the field of esthetics and shade matching has predominantly been conducted in Western populations. However, with increasing dental awareness and demand for esthetics in developing countries, such as the Indian subcontinent, research in the field of esthetics based on the local population has become the need of the hour.

This study was designed to determine the prevalence, satisfaction, and preference of tooth shades and their correlation with age, gender, and skin color among Indian individuals.

The null hypothesis was that there would be no difference in the prevalence, preference, and satisfaction of tooth shades between the age class, gender, and skin color patterns.

## Methods

### Study setting

A cross section of about 120 subjects visiting the outpatient Department of Prosthodontics and Crown and Bridge, of this Institution were randomly recruited for the study. Informed consent was obtained under a protocol reviewed and approved by the Institutional board.

### Pilot study and sample size calculation

A pilot study indicated a 56.52% satisfaction rate. Using a significance level of 5% and a minimum clinically important difference of 10%, the calculated sample size was 95. For even distribution among age and gender groups, the sample was increased to 120, employing matched quota sampling. While this method ensures balanced representation, we acknowledge that it may introduce selection bias, thereby limiting generalizability.

### Eligibility criteria

120 participants were recruited in this study using the following inclusion criteria:

• Presence of all six maxillary anterior teeth (canines to central incisors).
• No external discoloration or staining.
• Uniform shade across all six anterior teeth.

### Grouping

Participants were divided into eight groups based on gender and age: 20–30, 30–40, 40–50, and 50–60 years. These age brackets were selected based on esthetic transitions commonly observed in adults.

The color of their teeth was visually evaluated using the 3D Master shade Guide.

### Procedure

*Questionnaire*

Participants completed a demographic questionnaire, including satisfaction with their current tooth shade.

*Shade selection*

**Figure 1.**
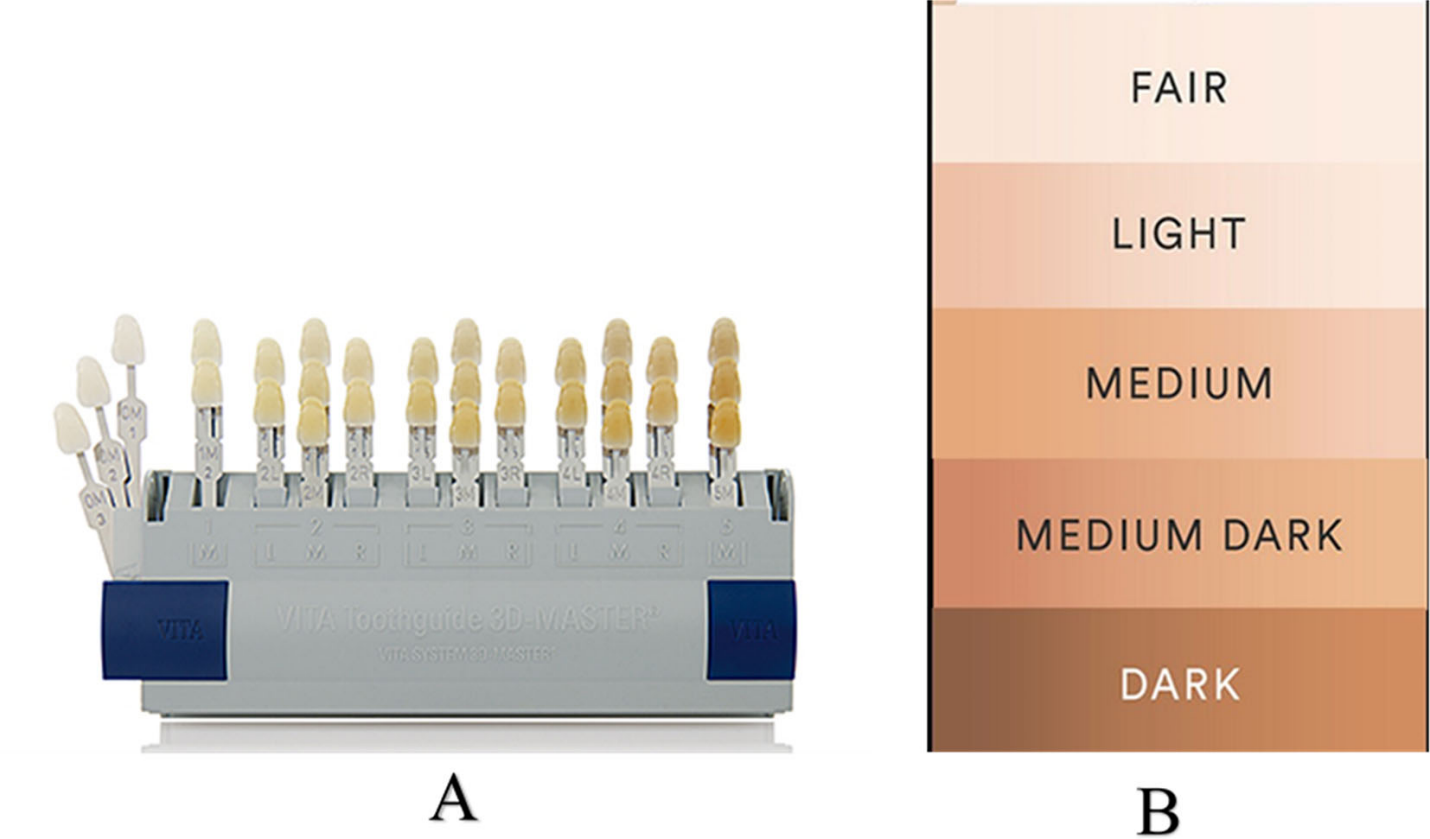
A - VITA Tooth-Guide 3D Master. B - L'Oreal skin shade guide.

The examiner was screened using the Ishihara test for color blindness. Shade selection was performed under natural lighting (northern sky exposure, approx. 2,000–5,000 lux), avoiding bright surroundings. Female participants were asked to remove lipstick. A light blue background was used during the examination. A thorough oral prophylaxis was performed, and shade matching was performed under moist conditions, at arm’s length, and at the operator’s eye level. The least conspicuous color was selected using a squint test. The VITA Tooth-Guide 3D Master (3D Master, VITA Zahnfabrik H. Rauter GmbH & Co. KG Postfach 1338 D-79704 Bad Säckingen) was used for both existing and preferred shade matching. Skin color was assessed using the L’Oréal skin shade guide on the inner forearm under natural light. ( Figure 1A & B).

### Data management and statistical analysis

Data were analyzed using IBM SPSS v20. Chi-square tests and logistic regression were used to examine correlations. Odds ratios (ORs) and 95% confidence intervals (CIs) were calculated to measure associations between demographic variables and tooth shade preference. A p-value < 0.05 was considered statistically significant.

## Results

A total of 120 participants were grouped based on their age and sex to evaluate the prevalence, satisfaction, and preference of tooth shade and their correlation with age, sex, and skin shade using questionnaires along with the VITA Tooth-Guide 3D Master shade guide and L’Oreal skin shade guide.

### Existing & preferred tooth shades

The most common existing tooth shade was Value 2 (52.5%). The least common was Value 0. The most preferred tooth shade was also Value 2 (58.33%), with 2L1.5 being the most liked specific shade ( Tables 1 & 2).

**Table 1.** Distribution of existing and preferred tooth shade values among participants (N = 120).

Tooth shade value	Participants with existing shade (%)	Participants preferring shade (%)
Value 0	0.0%	3.3%
Value 1	8.3%	20.0%
Value 2	52.5%	58.3%
Value 3	36.7%	17.5%
Value 4	2.5%	0.8%

**Table 2.** Most common preferred specific shades (Top 6).

Shade code (VITA 3D Master)	Number of participants (n)	Percentage (%)
2L1.5	21	17.5%
2M1	18	15.0%
1M1	15	12.5%
2R1.5	14	11.7%
1M2	9	7.5%
2M2	8	6.7%

Participants aged 41–50 years were significantly more likely to prefer shade Value 2 (OR = 2.67, 95% CI: 1.40–5.09, p < 0.001) ( Tables 3 & 4).

**Table 3.** Chi-square analysis of preferred tooth shade by age group (Value 2 preference).

Age group (years)	χ ^2^	df	p-value	Most-preferred shade (n, %)
20–30	4.42	4	0.352	Value 2 (14, 46.7%)
31–40	5.56	3	0.135	Value 2 (17, 56.7%)
41–50	—	2	< 0.001	Value 2 (22, 73.3%)
51–60	2.27	2	0.321	Value 2 (19, 63.3%)

A p-value < 0.05 was considered statistically significant. The 41–50 age group showed a statistically significant preference for Value 2 shades.

**Table 4.** Logistic regression of age and gender on preference for Value 2 shade.

Variable	Odds Ratio (OR)	95% Confidence Interval (CI)	p-value	Significance
Age 41–50 (vs. others)	2.67	1.40 – 5.09	< 0.001	**Significant**
Male (vs. Female)	1.23	0.68 – 2.21	0.480	Not Significant

**Figure 2.**
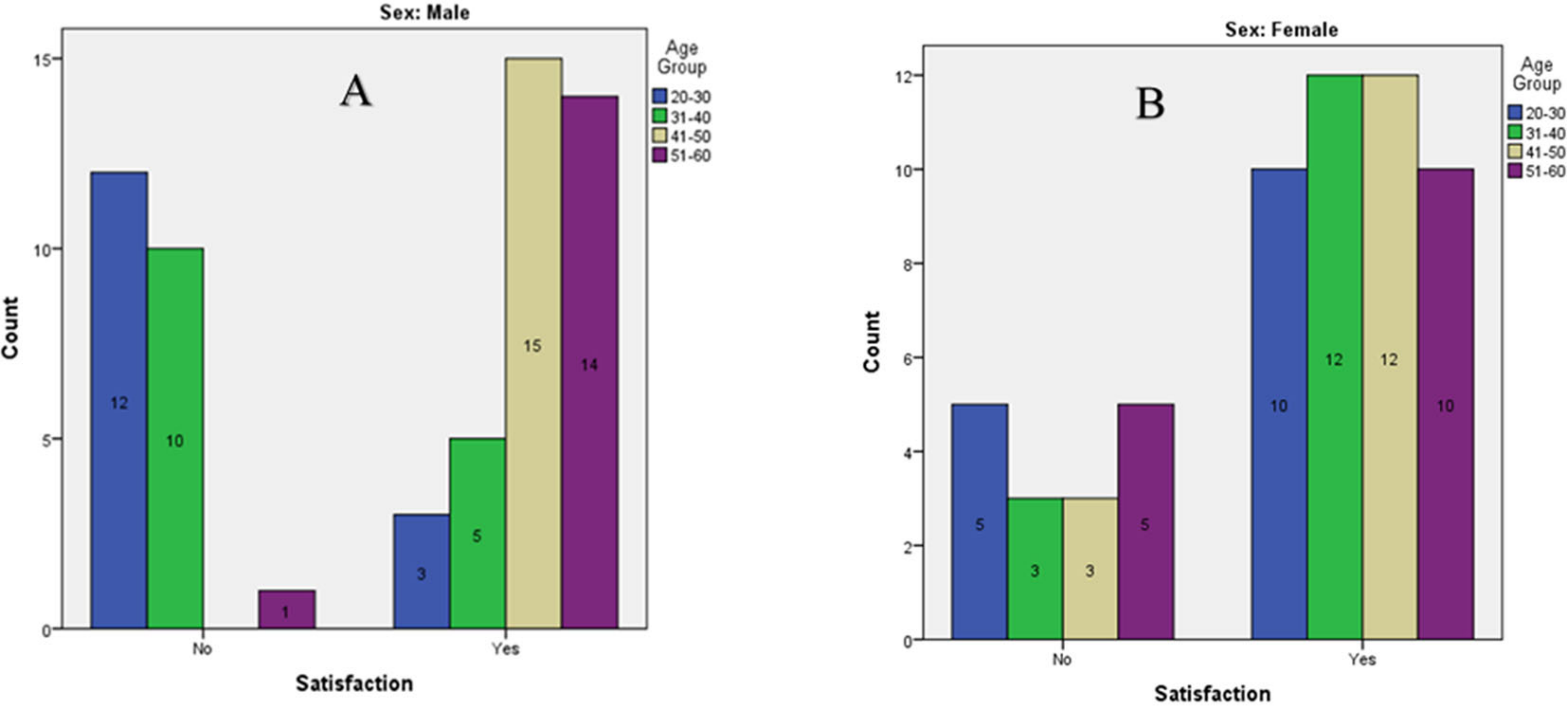
Bar graph of Satisfaction of tooth shade among participants of different age groups. A) Males. B) Females.

Chi-square tests confirmed significant variation in satisfaction levels across different groups (p < 0.001 for males). Satisfaction was highest among males in the 41–50 age group ( Tables 5, 6 & Figure 2).

**Table 5.** Comparison of satisfaction with existing tooth shades by gender and age group.

Gender	Age group (years)	Satisfied (n)	Not satisfied (n)	Total (n)
Female	20–30	10	5	15
Female	31–40	12	3	15
Female	41–50	12	3	15
Female	51–60	10	5	15
Total		**44**	**16**	**60**
Male	20–30	3	12	15
Male	31–40	5	10	15
Male	41–50	15	0	15
Male	51–60	14	1	15
Total		**37**	**23**	**60**

**Table 6.** Chi-square analysis of satisfaction by gender.

Gender	Chi-square value (χ ^2^)	Degrees of freedom (df)	p-value	Significance
Female	1.36	3	0.714	Not Significant
Male	31.80	3	< 0.001	**Significant**

A p-value < 0.05 is considered statistically significant. Satisfaction levels among males showed significant variation across age groups, particularly higher in older age brackets.

### Skin color

Although statistical analysis was performed to assess the association between skin color and tooth shade preference or satisfaction, no significant correlation was observed. These results are not shown in table form due to lack of statistical significance.

## Discussion

Shade selection remains a subjective process, particularly in edentulous patients lacking reference data. To ensure good esthetics, the attributes of color namely, hue, chroma and value must be correctly chosen. Amongst these attributes Value is most important since a small variation in chroma and hue will not be noticeable if value blends. ^16^

Methods of dental shade selection can be broadly categorized into visual and digital methods. Visual methods, popularly used include the use of stocks and custom shade guides. Digital methods include digital cameras, color-measuring software, colorimeters, spectrophotometers, and intraoral scanners. Despite developments in dental shade selection methods, shade selection remains a challenge that affects esthetic outcomes. ^17^

This challenge is augmented when patients are edentulous and lack any prior photographic or dental records of the shade of their original teeth, and conventional dental shade selection tools cannot be used.

There are several recommendations for selecting the shape and size of artificial teeth, which are supported by rationale and empirical data. ^18^^–^^21^

However, shade selection for completely edentulous patients follows vague and broad criteria and is therefore more subjective and arbitrary. These add complexities to complete dentures and full-mouth rehabilitation. ^18^^–^^23^

This study confirms that age and gender affect tooth shade satisfaction and preference, while skin color does not. These findings lead to a partial rejection of the null hypothesis. While age and gender demonstrated statistically significant associations with participants’ satisfaction and preference for tooth shades, no such relationship was observed with skin color. This supports the need for demographic-specific considerations during shade selection in clinical practice, particularly regarding age and gender.

The distribution tables reveal a consistent pattern in shade preference, with 58.3% of participants preferring Value 2 ( Table 1), and specific shades such as 2L1.5 and 2M1 being the most frequently selected ( Table 2). Chi-square analysis also revealed that this age group (41–50 years) demonstrated statistically significant variation in shade preference (p < 0.001), unlike other age groups ( Table 3). This reinforces the interpretation that middle-aged adults are more definitive and selective in their shade preferences. As age advances, people tend to become more involved in solving daily issues and are more concerned with greater challenges in life rather than their tooth color.

Logistic regression further confirms that individuals aged 41–50 years were significantly more likely to prefer Value 2, with an odds ratio of 2.67 (95% CI: 1.40–5.09), highlighting a clear age-related preference trend ( Table 4).

The comparison of satisfaction across age and gender groups ( Tables 5, 6 & Figure 2) showed that satisfaction with existing tooth shade was significantly higher in older male participants, particularly in the 41–50 and 51–60 year groups. Conversely, younger males (20–30 years) reported the lowest satisfaction levels. Females generally reported high satisfaction across all age groups, but the differences were not statistically significant. This finding aligns with similar studies done by Tin-Oo et al. and Maghaireh et al. ^14^^,^^15^

The preference for Value 2 aligns with findings in similar populations, reinforcing its reliability for clinical use.Comparisons with existing literature affirm similar trends in satisfaction and value preferences. ^24^^,^^25^ However, methodological differences may account for any contrasting outcomes in Western populations, where lighter shades are often more desirable. ^26^ These cultural nuances underscore the importance of regional studies to guide patient-specific esthetic decisions.

Our study thus emphasizes the need for culturally specific shade selection guidelines and supports a data-driven approach to enhancing esthetic outcomes in prosthodontic care.

These findings have practical clinical relevance. The clear preference for Value 2, combined with higher satisfaction among older age groups, suggests that this shade category is not only commonly desired but also more likely to meet patient expectations, especially in complete denture cases where pre-extraction records are unavailable. For younger or esthetically conscious patients who may prefer brighter shades, options such as tooth whitening or cosmetic restorations may be considered. As people age, their teeth tend to become darker, which is likely due to the formation of secondary dentin.

## Conclusions

Within the limitations of this study, the following conclusions can be drawn:

• Satisfaction with existing tooth shade increases with age, particularly among males aged 41–50.
• The most prefered tooth shades across all groups were of Value 2, according to the VITA Tooth-Guide 3D Master.

### Limitations of the study

This study had several limitations. The use of a matched quota sampling method, while ensuring balanced representation across age and gender, may introduce selection bias. The absence of randomization and blinding could also lead to observer-related bias in shade and skin color evaluation. Although our sample size of 120 was statistically adequate, a larger, more diverse cohort might yield additional correlations, particularly across broader skin tone categories. Furthermore, only one investigator performed the evaluations; although calibration was conducted, inter-observer reliability could not be assessed.

#### Key points

• In the absence of dental records, age and gender are useful guides for shade selection.
• Shades in the Value 2 category are broadly acceptable across demographics.
• Males in older age groups may be more satisfied with darker shades, while females prefer lighter shades regardless of age.

#### Ethical considerations

All observations were performed in conformity with the ethical standards of the Institutional Ethics Committee, Manipal College of Dental Sciences Mangalore, after receiving approval from the committee (ref:22007 dated:12/02/2022). Each individual assigned to participate in the study gave written informed consent to participate in the study.

## Data Availability

Figshare: prevalence, satisfaction and preference of tooth shades and their correlation with age, gender and skin shade: a cross sectional study, DOI: https://doi.org/10.6084/m9.figshare.24903114.v3. ^27^ This project contains the following:
• Raw data Data are available under the terms of the Creative Commons Attribution 4.0 International license (CC-BY 4.0). Figshare: prevalence, satisfaction and preference of tooth shades and their correlation with age, gender and skin shade: a cross sectional study, DOI: https://doi.org/10.6084/m9.figshare.24903114.v3. ^27^ This project contains the following:
• Questionnaire Data are available under the terms of the Creative Commons Attribution 4.0 International license (CC-BY 4.0). Figshare: prevalence, satisfaction and preference of tooth shades and their correlation with age, gender and skin shade: a cross sectional study, DOI: https://doi.org/10.6084/m9.figshare.24903114.v3. ^27^ This project contains the following:
• STROBE CHECK Data are available under the terms of the Creative Commons Attribution 4.0 International license (CC-BY 4.0).

## References

[1] Samorodnitzky-Naveh GR Geiger SB Levin L : Patients’ satisfaction with dental esthetics. *J. Am. Dent. Assoc.* 2007;138:805–808. 10.14219/jada.archive.2007.0269 17545270

[2] Jahangiri L Reinhardt SB Mehra RV : Relationship between tooth shade value and skin color: an observational study. *J. Prosthet. Dent.* 2002;87:149–152. 10.1067/mpr.2002.121109 11854669

[3] McAndrew R : *Contemporary Fixed Prosthodontics.* 3rd ed.Vol.29. Sage;2014 Dec; pp.328–329. 10.1093/ortho/29.4.328-a

[4] Azad A Ahmad S Zia M : Relationship of age, gender and skin tone to shades of permanent maxillary central incisors. *Pak. Oral Dent. J.* 2007;27:119–125.

[5] Moon RJ Millar BJ : Dental Aesthetics: A Study Comparing Patients’ Own Opinions with Those of Dentists. *Open J. Stomatol.* 2017 Apr 17 [cited 2022 Jan 6];07:225–233. 10.4236/ojst.2017.74016

[6] Marcucci B : A shade selection technique. *J. Prosthet. Dent.* 2003;89:518–521. 10.1016/S0022-3913(03)00076-3 12806332

[7] Hammad IA : Intrarater repeatability of shade selections with two shade guides. *J. Prosthet. Dent.* 2003;89:50–53. 10.1067/mpr.2003.60 12589286

[8] Zarb G Bolender CL : *Prosthodontic treatment for edentulous patients: complete dentures and implant-supported prostheses.* 12th ed. St. Louis: Mosby Inc.;2004; pp.190–207.

[9] Bauer J Vasilache I Schlegel AK : Esthetics and Psyche—Part 1 Assessment of the Influence of Patients’ Perceptions of Body Image and Body Experience on Selection of Existing Natural Tooth Color. *Int. J. Prosthodont.* 2012;25:36–43. 22259794

[10] Ajayi EO : Dental aesthetic self-perception and desire for orthodontic treatment among school children in Benin City, Nigeria. *Nig. Q. J. Hosp. Med.* 2011;21(1):45–49. 21913541

[11] Albashaireh ZSM Alhusein AA Marashdeh MM : Clinical assessments and patient evaluations of the esthetic quality of maxillary anterior restorations. *Int. J. Prosthodont.* 2009;22(1):65–71. 19260431

[12] Hamamci N Baaran G Uysal E : Dental Aesthetic Index scores and perception of personal dental appearance among Turkish university students. *Eur. J. Orthod.* 2009 Apr;31(2):168–173. 10.1093/ejo/cjn083 19126820

[13] Afshar MK Eskandarizadeh A Torabi M : Patient Satisfaction with Dental Appearance and Related FactorsA Cross Sectional Study. *J. Evol. Med. Dent. Sci.* 2019;8:3569–3574. 10.14260/jemds/2019/771

[14] Maghaireh GA AIzraikat H Taha NA : Satisfaction with Dental Appearance and Attitude toward improving Dental Esthetics among Patients attending a Dental Teaching Center. *J. Contemp. Dent. Pract.* 2016;17:16–21. 10.5005/jp-journals-10024-1796 27084857

[15] Tin-Oo MM Saddki N Hassan N : Factors influencing patient satisfaction with dental appearance and treatments they desire to improve aesthetics. *BMC Oral Health.* 2011 Feb 23;11:1–8. 10.1186/1472-6831-11-6 21342536 PMC3059271

[16] Hassel AJ Nitschke I Dreyhaupt J : Predicting tooth color from facial features and gender: results from a white elderly cohort. *J. Prosthet. Dent.* 2008;99:101–106. 10.1016/S0022-3913(08)60025-6 18262010

[17] Tabatabaian F Beyabanaki E Alirezaei P : Visual and digital tooth shade selection methods, related effective factors and conditions, and their accuracy and precision: A literature review. *J. Esthet. Restor. Dent.* 2021;33:1084–1104. 10.1111/jerd.12816 34498789

[18] Sharry JJ : *Complete denture prosthodontics.* 3rd ed. New York: McGraw-Hill;1974.

[19] Johnson DL Stratton RJ : *Fundamentals of removable prosthodontics.* Chicago: Quintessence;1980; pp.289–307.

[20] Pound E : Applying harmony in selecting and arranging teeth. *Dent. Clin. N. Am.* 1962;6:241–258.

[21] Landa LS : Practical guidelines for complete denture esthetics. *Dent. Clin. N. Am.* 1977;21:285–298. 10.1016/S0011-8532(22)03210-4 321275

[22] Heartwell CM Rahn AO : *Syllabus of complete dentures.* 3rd ed. Philadelphia: Lea & Febiger;1980; pp.293–306.

[23] Zarb GA Bolender CL Hickey JC : *Boucher’s prosthodontic treatment for edentulous patients.* 10th ed. St Louis: CV Mosby Co;1990; pp.330–342.

[24] Esan TA Olusile AO Akeredolu PA : Factors Influencing Tooth Shade Selection for Completely Edentulous Patients. *J. Contemp. Dent. Pract.* 2006;7:80–87. 10.5005/jcdp-7-5-80 17091143

[25] Al-Dwairi Z Shaweesh A Kamkarfar S : Tooth shade measurements under standard and nonstandard illumination and their agreement with skin color. *Int. J. Prosthodont.* 2014;27:458–460. 10.11607/ijp.3826 25191889

[26] Vadavadagi SV Kumari KV Choudhury GK Prevalence of Tooth Shade and its Correlation with Skin Colour - A Cross-sectional Study. J. Clin. Diagn. Res. 2016 Feb;10(2): ZC72-4. 10.7860/JCDR/2016/16918.7324 PMC4800657 27042590

[27] Bajantari P : Prevalence, satisfaction, and preference of tooth shades and their correlation with age, gender, and skin shade: a cross-sectional study.Dataset. *figshare.* 2023. 10.6084/m9.figshare.24903114.v3 PMC12203042 40585878

