# Supplementary material for: Prevalence, satisfaction and preference of tooth shades and their correlation with age, gender and skin color : A cross sectional study
Source: F1000Res. 2025 Jul 14;13:1076. Originally published 2024 Sep 19. [Version 3] doi: 10.12688/f1000research.146428.3 (PMC12203042; doi:10.12688/f1000research.146428.3)
Supplement: Peer review history

# Reviewer report: Reviewer response for version 2

**DOI:** 10.5256/f1000research.176521.r394635

**Published:** 2025-06-26

**Authors:** Leszek Szalewski

**Affiliations:** 1 Medical University of Lublin, Lublin, Poland

The manuscript titled *"Prevalence, satisfaction and preference of tooth shades and their correlation with age, gender and skin shade: A cross-sectional study"* addresses an important topic in contemporary prosthodontics: the subjective and multifactorial nature of tooth shade selection and its relationship with demographic variables. The study is relevant and timely, particularly within the context of increasing esthetic awareness in the Indian population.

 The abstract provides a comprehensive summary of the research; however, it includes a somewhat misleading statement suggesting that difficulties in shade selection persist “despite the presence of dental records or photographs.” In fact, such difficulties primarily arise from the inherent subjectivity of visual shade selection methods. It would be more precise to attribute this challenge to the method used rather than the availability of historical data.

 The introduction is well-structured and touches on key background concepts, but it would benefit from greater linguistic consistency—particularly with regard to the spelling of "esthetic" versus "aesthetic" and "color" versus "colour." Several short paragraphs could be merged to improve flow and coherence. Some of the cited references are dated and could be supplemented with more recent literature to enhance the scientific rigor.

 The methods section provides a general overview of the sampling and procedures used, including the use of the VITA Tooth-Guide 3D Master and the L’Oréal skin shade guide. However, several methodological concerns merit attention. The choice of a matched quota sampling technique introduces potential bias, despite its utility in ensuring equal gender and age group representation. The study lacks a clear justification for the selected age brackets, and the protocol for tooth shade and skin shade evaluation could be described in more detail—particularly regarding lighting conditions, calibration of observers, and steps to minimize intra- and inter-observer variability.

 The statistical analysis is broadly appropriate, employing Chi-square tests and logistic regression. Nonetheless, key details—such as confidence intervals or odds ratios—are not provided, and Table 2 lacks clarity in explaining what was statistically significant. Some aspects of the presentation would benefit from clearer explanations and more consistent terminology (e.g., “desired” tooth shade rather than “wanted”).

 The discussion section is underdeveloped. Rather than offering an interpretive analysis of the findings in the context of existing literature, it reads as a list of factual observations. The authors miss the opportunity to explore the clinical implications of their results more deeply. For instance, while the preference for shade Value 2 is clearly demonstrated, the discussion lacks a thorough exploration of how this might inform clinical protocols, especially in edentulous patients.

 The limitations section is appropriate, though brief. It would be helpful to elaborate on the potential effects of selection bias, the relatively small sample size, and the lack of blinding or randomization.

Is the work clearly and accurately presented and does it cite the current literature?

Yes

If applicable, is the statistical analysis and its interpretation appropriate?

Partly

Are all the source data underlying the results available to ensure full reproducibility?

Partly

Is the study design appropriate and is the work technically sound?

Partly

Are the conclusions drawn adequately supported by the results?

Partly

Are sufficient details of methods and analysis provided to allow replication by others?

Partly

Reviewer Expertise:

prosthetics, dental photography, dental education,

I confirm that I have read this submission and believe that I have an appropriate level of expertise to confirm that it is of an acceptable scientific standard, however I have significant reservations, as outlined above.

# Reviewer report: Reviewer response for version 2

**DOI:** 10.5256/f1000research.176521.r369658

**Published:** 2025-03-27

**Authors:** Stephen Westland

**Affiliations:** 1 School of Design, University of Leeds, Leeds, UK

Many thanks. I just read the revised manuscript and I have seen the response to the comment. On this basis I am happy to recommend the manuscript be accepted.

Is the work clearly and accurately presented and does it cite the current literature?

Yes

If applicable, is the statistical analysis and its interpretation appropriate?

Yes

Are all the source data underlying the results available to ensure full reproducibility?

Yes

Is the study design appropriate and is the work technically sound?

Yes

Are the conclusions drawn adequately supported by the results?

Partly

Are sufficient details of methods and analysis provided to allow replication by others?

Yes

Reviewer Expertise:

NA

I confirm that I have read this submission and believe that I have an appropriate level of expertise to confirm that it is of an acceptable scientific standard.

# Reviewer report: Reviewer response for version 1

**DOI:** 10.5256/f1000research.160517.r339854

**Published:** 2024-12-12

**Authors:** Dr. Gbenga Emmanuel Adebayo

**Affiliations:** 1 Department of Dental Services, Federal Medical Centre, Ebute-metta, Nigeria

This article focused on the prevalence, satisfaction of patients and their preference for tooth shade and how it correlate with their age gender and skin color. This is a very good research topic as it addresses the aesthetic need and satisfaction of patient.  The demand of patient for dental prosthesis that meet their aesthetic quest is on the rise, and tooth shade matching is a significant factor in meeting this demand. However, having read through the article thoroughly, there are things I observed and these are pointed out under the different sections of the article as follows:

**Title**

 The title of the research is clear and objectives well captured, although “skin color” would have been more appropriate than “skin shade”

**Abstract**

 The author made mention in the abstract that in the absence of teeth and dental photograph, tooth shade selection becomes challenging and subjective. But I want to say that whether teeth and dental photograph are available or not, tooth shade matching is still challenging and subjective if conventional visual method of tooth shade selection is used. Subjectivity of shade selection primarily has to do with the method of shade matching used, whether it is visual or machine aided such as spectrophotometer. I feel the sentence can be rephrased for better understanding.

**Introduction**

 Introduction seems good and past studies were referenced. However, the author is not consistent with the use of “esthetic and ‘aesthetic”, he should have stuck to one. Also, the introduction has too many small paragraphs that can be combined.

**Method**

 - Sampling technique used for this study not appropriate in my own opinion as it might have introduced bias, although it has the advantage of making grouping into equal male and female easy.

 - Procedure for the study is not detailed. For example, the tooth shade matching steps was not stated clearly, were the investigators screened for eye anomaly or not, was it done under natural light or not, were the teeth wet or dry etc. For the skin shade matching, which part of the body skin was used, how was the matching done etc.

 - Age bracket of the participants and the reason should have been included in the selection criteria not just showing it in the grouping.

 - The description of the shade value should have been presented for better understanding of the results.

**Result**

 Result well presented in my own view and breaking it into sections made it clearer

**Discussion**

 Discussion is not adequate, nearly repetition of the result earlier presented, not much reference was made to other studies to corroborate the findings. I feel the discussion can be worked on to make it more robust.

**Conclusion**

 Well concluded

Is the work clearly and accurately presented and does it cite the current literature?

Partly

If applicable, is the statistical analysis and its interpretation appropriate?

I cannot comment. A qualified statistician is required.

Are all the source data underlying the results available to ensure full reproducibility?

Yes

Is the study design appropriate and is the work technically sound?

Partly

Are the conclusions drawn adequately supported by the results?

Yes

Are sufficient details of methods and analysis provided to allow replication by others?

Partly

Reviewer Expertise:

Aesthetic Restorative Dentistry, Endodontics and Dental implantology

I confirm that I have read this submission and believe that I have an appropriate level of expertise to confirm that it is of an acceptable scientific standard, however I have significant reservations, as outlined above.

# Response

**Published:** 2024-12-31

**Authors:** Dr Prashanth Bajantri

**Affiliations:** Prosthodontics crown and bridg, Manipal University, Mangalore, Karnataka, India

We thank both the reviewers and the Editorial for the time and effort invested in our manuscript and we are most happy to make the changes suggested by the reviewers.

 All the recommendations made by the reviewers have been humbly accepted and the necessary rectifications have been  made in the document.

 However, if the reviewers  feel the need for any more corrections we are happy to do so

 kind regards,

 Shobha Rodrigues

 shobha.j@manipal.edu

 corresponding Author

# Reviewer report: Reviewer response for version 1

**DOI:** 10.5256/f1000research.160517.r339847

**Published:** 2024-11-28

**Authors:** Stephen Westland

**Affiliations:** 1 School of Design, University of Leeds, Leeds, UK

- This is an interesting manuscript that explores the relationship between prevalence, satisfaction and preference for shades and age, gender and skin shade. 
- I believe that the work was carried out with Indian participants. With that in mind I think it would be sensible to specify this in the abstract.
- In the Methods section a Z value of Z=-1.95996398454005 is reported. I don't think this number of decimal places is justified. I suggest this be changed to 1.96.
- In terms of the pilot study conclusions, are the authors satisfied that 120 participants in total should be recruited rather than 120 in each of the age age/gender categories?
- Under Eligibility Criteria point 2 should be changed from 
  - "Teeth should be no external discoloration or staining" to
- "Teeth should have no external discoloration or staining."
- In the Grouping section, shouldn't the table have a title, e.g. Table 1: blah blah.
- Under Procedure the authors write:
  - "The investigators then selected the most common tooth shade among the maxillary anterior teeth" Is this really what the mean? In which case, what was the most common tooth shade. I think they might mean to write that they selected the tooth shade that best matched the participant's teeth.
- Under Wanted tooth shades, I think this would be better expressed as Desired tooth shades. 
- Under wanted shade section, I do find it confusing. For example, in Table 1 it is evident that Value 2 is most prevalent and most preferred. Later, in the statistical analysis in Table 2 it is reported that there is significance for the the 41-50 age group. But what is significant? It is not clear.
- The way the Discussion is presented is not very engaging. It is simply a list of observations with very little discussion involved. 
- Ultimately, although satisfaction and preference is interesting, I think prevalence is most important. The finding is that most people have teeth of Value 2. Is there any correlation of tooth shade prevalence with age or gender? 
- I think a clearer Discussion is needed. Factual observations should be moved to the Results section. The list of observations in the Discussion is too long. A proper discussion of what the results mean clinically is needed.

Is the work clearly and accurately presented and does it cite the current literature?

Yes

If applicable, is the statistical analysis and its interpretation appropriate?

Yes

Are all the source data underlying the results available to ensure full reproducibility?

Yes

Is the study design appropriate and is the work technically sound?

Yes

Are the conclusions drawn adequately supported by the results?

Partly

Are sufficient details of methods and analysis provided to allow replication by others?

Yes

Reviewer Expertise:

Specialist in colour and appearance of teeth

I confirm that I have read this submission and believe that I have an appropriate level of expertise to confirm that it is of an acceptable scientific standard, however I have significant reservations, as outlined above.
